# Rectus sheath hematoma in pregnancy: a case report with literature review

**DOI:** 10.3389/fmed.2025.1670254

**Published:** 2026-01-05

**Authors:** Yongbing Guo, Yan Gao, Huixia Yang, Yu Sun

**Affiliations:** Peking University First Hospital, Beijing, China

**Keywords:** rectus sheath hematoma, pregnancy, transfusion, ultrasonography, case report

## Abstract

Rectus sheath hematoma (RSH) is a rare cause of abdominal pain during pregnancy. In our case, a healthy pregnant woman presented with right upper abdominal pain at 33 weeks of gestation. Ultrasound showed a heterogeneous hypoechoic mass in the abdominal wall, which was suspected of being a rectus sheath hematoma. After half a day of conservative therapy, the progressive decrease in hemoglobin level suggested active bleeding into her rectus sheath. Consequently, surgery was initiated under general anesthesia. During the operation, we found that the right musculus rectus abdominis was ruptured and actively bleeding. The hematoma was removed, and the broken end of the bleeding rectus abdominis muscle was ligated. After the operation, a blood transfusion was given, and the patient was discharged from the hospital as scheduled. A healthy newborn was delivered by elective cesarean section at 39 weeks of gestation. We present this case to enhance the recognition of RSH during pregnancy. Although relatively rare, this condition manifests itself with an acute onset and severe symptoms. Timely and accurate diagnosis and management may effectively reduce the incidence of preterm delivery.

## Introduction

Rectus sheath hematoma (RSH) refers to hemorrhage into the rectus sheath. It most often results from trauma to the inferior or superior epigastric arteries and their branches or, less commonly, from a tear of the rectus abdominis muscle (1). Anticoagulation, coughing, exertion, exercise, hypertension, obesity, previous abdominal surgery, subcutaneous injection, and trauma are also contributing factors for RSH (2). For special patients, such as the elderly with multiple comorbidities, patients with abdominal rectus sheath hematoma can achieve Positive results can be obtained by avoiding unnecessary surgical procedures with correct diagnosis and treatment approaches (3).

RSH is a rare cause of abdominal pain during pregnancy. Although RSH is a relatively rare occurrence in pregnancy, improper clinical management can pose a significant risk of preterm labor and potential maternal mortality, which is reported to cause a maternal mortality rate of 13% and a perinatal morbidity rate of 50% (4–6). Early diagnosis helps reduce the rate of unnecessary preterm birth. We describe a case of rectus sheath hematoma resulting from a rectus abdominis tear during pregnancy, highlighting the diagnostic difficulties and emphasizing the critical need for timely intervention. Finally, the patient’s condition was successfully managed surgically and resulting in a full-term delivery. The discussion will address the risk factors, clinical presentation, diagnosis, and management of RSH during pregnancy.

## Case report

A 33-year-old gravida 3 para 2 woman with an uncomplicated 33-week singleton pregnancy was transferred to our obstetric emergency unit following a 10-day history of cough and acute exacerbation of right-sided abdominal pain over 4 h. The patient described severe, constant, non-radiating pain in the upper abdomen, exacerbated by coughing and movement. There was no reported fever, chills, uterine contractions, vaginal bleeding, trauma, falls, or anticoagulant use. Her medical history included surgical repair of a ventricular septal defect and two prior cesarean deliveries. She had no history of uterine fibroids, ovarian masses, or recurrent abdominal pain.

**Figure 1:**
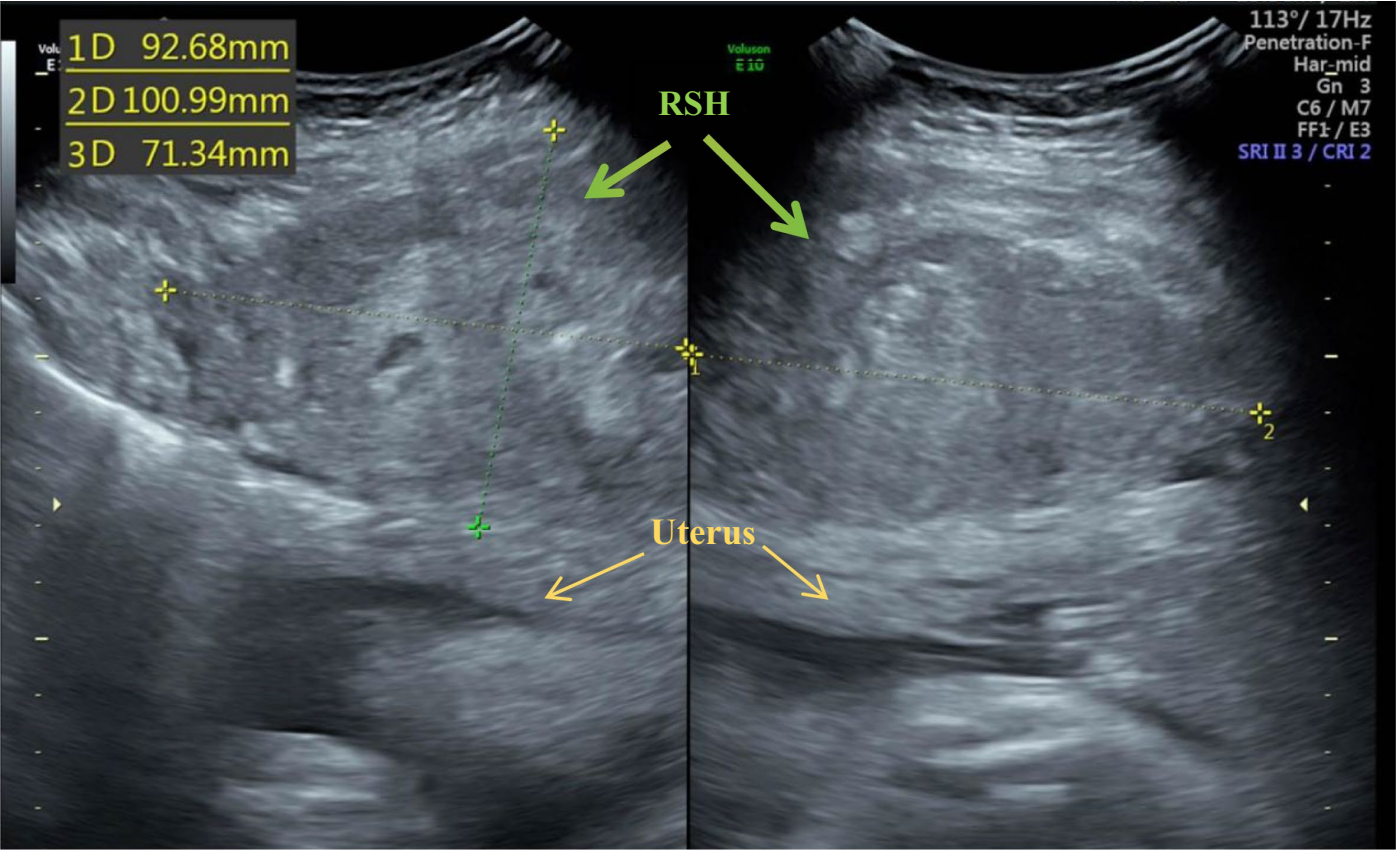
Ultrasonographic image shows the rectus sheath hematoma (RSH, green arrow) and the uterus (yellow arrow).

On admission, her vital signs were stable. Fetal cardiotocography revealed a reactive non-stress test with no uterine activity. Laboratory findings were notable for a hemoglobin level of 101 g/L. Ultrasound examination demonstrated a fetus with biometric measurements appropriate for gestational age. However, a well-defined hypoechoic mass (93 × 101 × 73 mm) was observed beneath the right fascia, with no apparent sonographic evidence of placental abruption identified (Figure 1). In response to the sonographically diagnosed rectus abdominis hematoma, an urgent multidisciplinary consultation was held with specialists from cardiology, pulmonology, general surgery, neonatology, and obstetrics to devise a treatment plan. Conservative management was chosen as the initial therapy due to the patient’s stable condition and the immaturity of the fetus.

Management was initiated with intramuscular dexamethasone for fetal lung maturation, intravenous tranexamic acid (1 g) for hemostasis, and broad-spectrum antibiotics for prophylaxis. However, within 30 min, the patient reported worsening abdominal pain. The patient’s BP monitor revealed a significant drop in blood pressure. A repeat complete blood count showed a decrease in hemoglobin from 96 g/L to 78 g/L, indicating active hemorrhage. Given the failure of conservative measures, emergent surgical exploration was decided. Continuous fetal monitoring was maintained throughout, with readiness for emergent cesarean delivery if indicated.

A large hematoma, containing a significant volume of blood clots and active hemorrhage, was identified beneath the rectus abdominis muscle upon surgical exploration through an incision along the anterior aspect of the rectus sheath. Subsequent evacuation of the hematoma exposed the source of bleeding: a 2 cm spontaneous rupture of the muscle with active hemorrhage at the transected end. The ruptured muscle ends were sutured and ligated to achieve complete hemostasis. Hemostatic gauze was applied, and a subfascial drain was placed prior to closure with tension-reducing sutures. The patient was transfused with 4 units of packed red blood cells, 400 mL of plasma, and 120 mL of autologous blood. Her postoperative hemoglobin level stabilized at 90 g/L. The remainder of her pregnancy was uneventful. She underwent a scheduled cesarean delivery at 39 weeks of gestation. At 1-year follow-up, the mother had fully recovered without sequelae, and the neonate exhibited growth parameters consistent with the standard percentiles.

## Discussion

Rectus sheath hematoma (RSH) during pregnancy is a rare condition, with its incidence yet to be well-documented in the literature (7). It typically occurs in the third trimester, often triggered by factors such as coughing, trauma, or adhesions, which can cause rupture of the epigastric vessels or their branches due to sudden muscular contraction or stretching (8, 9). Additionally, physiological vascular engorgement of the abdominal wall during pregnancy, combined with mechanical stretching from the expanding uterus, increases susceptibility to spontaneous vessel rupture and subsequent hematoma formation (10). In the present case, the patient had a recent history of coughing and two previous cesarean deliveries, which likely led to adhesions between the abdominal muscles and the peritoneum. The forceful and repetitive coughing may have caused overstretching and rupture of muscle fibers, resulting in bleeding and hematoma formation behind the rectus abdominis muscle. In contrast to the well-documented etiology involving the inferior epigastric artery and its branches, the operative findings in this case revealed bleeding solely from the rectus abdominis stump (11, 12). This may be attributed to significant adhesions involving the muscle, fascia, and peritoneum secondary to previous surgery, and no discrete bleeding vessel was found.

Clinically, RSH in pregnancy typically presents with acute abdominal pain and may be accompanied by fever, dizziness, vomiting, or abdominal discomfort (13–15). A large hematoma can lead to complications such as hypotension, anemia, or even abdominal compartment syndrome (16). Below the arcuate line, where the posterior layer of the rectus sheath is absent, a hematoma may irritate the peritoneum and elicit signs of peritoneal irritation, complicating the diagnosis (3, 17). Previous reports indicate that RSH can be misdiagnosed as ovarian torsion, degenerating fibroids, hemolysis, elevated liver enzymes, and low platelets (HELLP) syndrome, acute fatty liver of pregnancy, or uterine rupture (18–20).

Ultrasonography demonstrates a sensitivity of 75–95% in diagnosing RSH, while computed tomography (CT) achieves 95–100% accuracy (21, 22). Magnetic resonance imaging (MRI) can also be of value in the diagnosis of rectus sheath hematomas. Based on CT findings, Berna et al. (23) proposed a classification system for RSH: Type I: An intramuscular hematoma with muscle enlargement that appears oval- or spindle-shaped and contains hyperattenuating areas; unilateral; no fascial dissection. Type II: Intramuscular hematoma with blood extending between the muscle and the transversalis fascia; can be unilateral or bilateral and does not include the prevesical space. The attenuation values for liquid blood are approximately 45 HU, while those for clotted components are 85 HU. Type III: Hematoma extends beyond the transversalis fascia into the peritoneum or prevesical space. Patients with Type I RSH are generally hemodynamically stable. In contrast, Types II and III may present with instability, manifested by altered mental status, hypotension, tachycardia, acute kidney injury, and a sudden or progressive drop in hemoglobin or hematocrit.

RSH in pregnancy can be self-limiting. In hemodynamically stable patients, conservative management—including rest, analgesia, anti-inflammatory medications, fluid resuscitation, and transfusion if necessary—is the initial approach (24). In cases of rapidly expanding hematoma accompanied by hemodynamic instability, angiographic embolization or surgical intervention should be considered. In non-pregnant patients, angiographic embolization may be regarded as first-line treatment. For pregnant patients, angiographic embolization may be used as a salvage option if surgical management fails, and it is considered relatively safe during pregnancy (18, 25). Surgical management involves hematoma evacuation and hemostasis, though identifying the bleeding source can be challenging due to the vascular anatomy (16). A paramedian or midline incision is typically used to access the rectus sheath. After achieving hemostasis, the hematoma is evacuated, the cavity irrigated, and a closed suction drain may be placed. In this case, the patient failed to respond to initial conservative management and developed hemodynamic instability, prompting urgent surgical exploration. Hemostasis was successfully achieved, and the patient was subsequently delivered at term. A healthy newborn was delivered by elective cesarean section at 39 weeks of gestation.

## Conclusion

We present this case to enhance the recognition of RSH during pregnancy. Although relatively rare, this condition manifests itself with an acute onset and severe symptoms. Timely and accurate diagnosis and management may effectively reduce the incidence of preterm delivery. Awareness of this condition in pregnancy is important since RSH is often misdiagnosed and since there are high associated perinatal (fetal and neonatal) and maternal morbidity and mortality rates.

## Data Availability

The original contributions presented in the study are included in the article/supplementary material, further inquiries can be directed to the corresponding author.
